# Choledochal Cyst Associated With Polycystic Kidney Disease: Report of a Case

**DOI:** 10.1155/1999/38936

**Published:** 1999-04

**Authors:** T. Hasegawa, M. Kim, Y. Kitayama, K. Kitamura, T. Hiranaka

**Affiliations:** 1 Department of Surgery Shirasagi Hospital 7-11-23 Kumada Higashisumiyoshiku Osaka-City 546 Japan; 2 Department of Pediatric Surgery Osaka University Medical School 2-2 Yamadaoka Suita City Osaka 565 Japan

## Abstract

We report a very rare case of type I choledochal cyst associated with a polycystic kidney disease. A 48- year-old female had been dependent on hemodialysis for chronic renal failure due to polycystic kidney disease and was incidentally diagnosed to have a dilated common bile duct by an ultrasonography. An endoscopic retrograde cholangiopancreatography showed a spindle-shaped, dilated common bile duct (type I choledochal cyst) without visualization of the pancreatic duct. She underwent a resection of the choledochal cyst. Intraoperative cholangiography showed no reflux of contrast medium into the pancreatic duct. Amylase level of the aspirated bile from the bile duct was not elevated. In the case of choledochal cyst combined with renal fibropolycystic disease, pancreaticobiliary maljunction may not contribute to the etiology of choledochal cyst. In such cases, management of choledochal cyst is still controversial and requires further discussion.


*HPB Surgery*, 1999, Vol. 11, pp. 185-190
Reprints available directly from the publisher
Photocopying permitted by license only

© 1999 OPA (Overseas Publishers Association) N.V.
Published by license under
the Harwood Academic Publishers imprint,
part of The Gordon and Breach Publishing Group.
Printed in Malaysia.

*Case Report*

### Choledochal Cyst Associated with Polycystic Kidney Disease: Report of a Case

T. HASEGAWA*, M. KIM, Y. KITAYAMA, K. KITAMURA and T. HIRANAKA

*Department of Surgery, Shirasagi Hospital, 7-11-23 Kumada, Higashisumiyoshiku, Osaka-City, Japan 546*

*(Received 30 July 1997; In final form 20 March 1998)*

**We report a very rare case of type I choledochal cyst associated with a polycystic kidney disease. A 48-year-old female had been dependent on hemodialysis for chronic renal failure due to polycystic kidney disease and was incidentally diagnosed to have a dilated common bile duct by an ultrasonography. An endoscopic retrograde cholangiopancreatography showed a spindle-shaped, dilated common bile duct (type I choledochal cyst) without visualization of the pancreatic duct. She underwent a resection of the choledochal cyst. Intraoperative cholangiography showed no reflux of contrast medium into the pancreatic duct. Amylase level of the aspirated bile from the bile duct was not elevated. In the case of choledochal cyst combined with renal fibropolycystic disease, pancreaticobiliary maljunction may not contribute to the etiology of choledochal cyst. In such cases, management of choledochal cyst is still controversial and requires further discussion.**

*Keywords:* Dilatation of the bile duct, choledochal cyst, polycystic kidney disease, fibropolycystic disease, pancreaticobiliary maljunction

### INTRODUCTION

Choledochal cyst (dilatation of the bile duct) is thought to be a type of hepatobiliary fibropolycystic disease [1-6] and many cases of choledochal cyst have been recognized to be caused by pancreaticobiliary maljunction [7-11]. Although polycystic kidney disease, renal fibropolycystic disease, is often associated with polycystic liver disease and Caroli's intrahepatic biliary dilatation, [1,2,4,5], it has been extremely rarely reported to be associated with choledochal cyst [3-6,12].

We describe a 48-year-old female with a rare association of Type I choledochal cyst [11] and polycystic kidney disease without pancreaticobiliary maljunction.

### CASE REPORT

At the age of 34 years, a female patient developed renal failure due to polycystic kidney

*Address for correspondence: Department of Pediatric Surgery, Osaka University Medical School, 2-2 Yamadaoka, Suita City, Osaka, Japan 565; Tel.: +81-6-879-3753; Fax: +81-6-879-3759.

disease detected by abdominal ultrasonography and computerized tomography. She had been dependent on hemodialysis 3 times a week since then. Her daughter also has polycystic kidney disease. At the age of 48 years, she suffered from right lateral abdominal pain and hematuria. Abdominal ultrasonography showed rupture of a cyst in the kidney and incidentally showed a dilated common bile duct. Until then she had not developed nausea, vomiting, jaundice or right hypochondralgia. Retrospective evaluation of the previous computerized tomography had shown the persistent dilatation of the common bile duct of the same size since the initial examination. Laboratory data were as follows: serum total bilirubin 0.23 mg/dl, direct bilirubin 0.06 mg/dl, aspartate aminotransferase 15 U/L, alanine aminotransferase 5 U/L, alkaline phosphatase 4.6 King Armstrong U/L, lactate dehydrogenase 240 U/L, gamma-glutamyl-trans-peptidase 8 U/L, cholinesterase 3340 U/L, carbohydrate antigen 19-9 71 U/ml (control <37), thymol turbidity test 3.6 U, zinc sulfate turbidity test 9.8 U, amylase 161 U/L, creatinine 9.5 mg/dl, blood urea nitrogen 47.0 mg/dl.

For further examination, an endoscopic retrograde cholangiopancreatography was performed and showed a spindle-shaped, dilated common bile duct and mildly dilated intrahepatic bile duct without any filling defect, which was classified as Type I choledochal cyst [11] (Fig. 1). The pancreatic duct was not visualized. However, pancreaticobiliary maljunction could

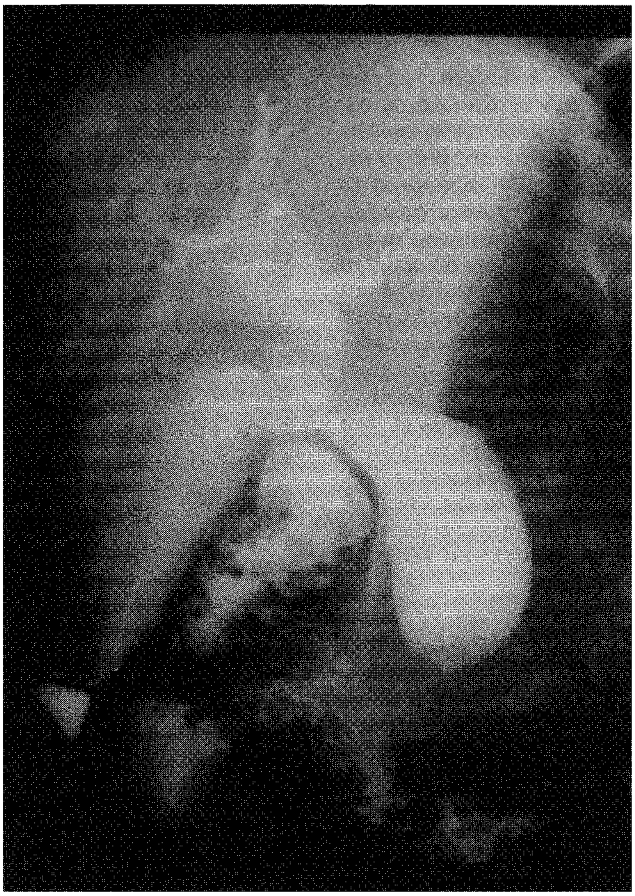

FIGURE 1 Endoscopic retrograde cholangiopancreatography shows a spindle-shaped, dilated common bile duct and dilatation of common hepatic duct and intrahepatic bile duct. Maximum diameter is about 3 cm.

not be completely denied at that time. We explained the necessity of operation to the patient, because persistence of the choledochal cyst might lead to infection, stone formation or malignant change in the biliary system. Subsequently, she underwent a cholecystectomy, resection of the choledochal cyst and hepaticojejunostomy with Roux-en Y loop. There was no stone or tumor formation. Intraoperative cholangiography showed no reflux of contrast medium into the pancreatic duct. Amylase level of the aspirated bile from the gall bladder and choledochal cyst was 25 and 26 U/L, respectively. Histology of the resected choledochal cyst wall showed mild chronic inflammation with dense collagen tissue. There was only a small number of glandular cavities. No malignant change was detected. The postoperative course was uneventful. The intrahepatic bile ducts have been gradually decreasing in size.

### DISCUSSION

Polycystic liver disease and polycystic kidney disease is a typical combination of fibropolycystic disease [1,2,4,5]. However, association of choledochal cyst and polycystic kidney disease has been extremely rare. The first report was done by Berenguer [12], 1976. In Chait's [6] report in 1994, he described that this combination had been seen only in 4 reports till that time [3–6, 12]. We could not find any other report by now. Table I summarizes 5 cases of these previous reports and the present case. The age at diagnosis of the choledochal cyst in these 6 cases ranged from 16 to 74 years. Of these 6 cases, 3 were Type V (Caroli's disease) and 2 were Type IV choledochal cyst [11]. The present case is the only one associated with Type I choledochal cyst. Nausea, vomiting, jaundice, fever, hepatomegaly or abdominal pain were the initial symptoms, while 2 cases including the present case were diagnosed incidentally at the evaluation of the other lesions. Three cases had additional hepatobiliary fibropolycystic disease, such as multiple liver cysts, or submucosal cysts in the gall bladder or the common bile duct.

Different types of choledochal cyst have different postulated etiologies. Junction of the biliary system and pancreatic duct outside the duodenal wall has been demonstrated to be the major cause of Type I choledochal cyst [7–11]. In the present case, however, long common channel or reflux of contrast medium from the bile duct into the pancreatic duct was not visualized by preoperative endoscopic retrograde cholangiopancreatography or intraoperative cholangiography. Amylase level of the bile in the biliary system was not elevated. There was only a small number of glandular cavities in the resected choledochal cyst wall. Therefore, pancreaticobiliary maljunction does not seem to have been associated in the present case. On the other hand, choledochal cyst has been reported to be one of the hepatobiliary forms of fibropolycystic disease, which is a systemic disease entity and includes renal forms, such as polycystic kidney disease, medullary sponge kidney and tubular ectasia [1,2]. The other hepatobiliary fibropolycystic disease includes polycystic liver disease, Caroli's intrahepatic biliary dilatation, congenital hepatic fibrosis (micropolycystic disease), microhamartoma, *etc.* [1,2]. The etiology of hepatobiliary fibropolycystic disease has been considered to be pancreaticobiliary maljunction or malformation of the embryonal ductal biliary plate [2,6]. In cases without evidence of pancreaticobiliary maljunction as in the present case, the other factors like malformation of the ductal biliary plate may contribute to the etiology of choledochal cyst.

Surgical resection of the choledochal cyst in cases associated with polycystic kidney disease is still controversial. In Chait's report [6], choledochotomy and cholecystectomy was performed because of stone formation in the biliary system. In Jordan's report [4], the patient had recurrent chronic cholecystitis, but died of sepsis after cholecystectomy. In the present case, the common bile duct had been persistently dilated for at least 12 years and we selected surgical

TABLE I Reported cases of choledochal cyst with polycystic kidney disease

| Case | Age | Symptom | Type | Complication | Treatment | Outcome | Author | Year |
|---|---|---|---|---|---|---|---|---|
| 1 | 16 | Fever, jaundice, hepatomegaly | V | Portal hypertension | Cholecystectomy, splenectomy | dead | Berenguer | 1976 |
| 2 | 31 | Jaundice | IV | None | unknown | unknown | Waldron | 1984 |
| 3 | 74 | None | V | Multiple liver cysts | unknown | unknown | Takehara | 1989 |
| 4 | 47 | Nausea, vomiting, rt.hypochondralgia | V | Recurrent cholecystitis, multiple liver cysts | Cholecystectomy | dead | Jordan | 1989 |
| 5 | 69 | Nausea, epigastric pain | IV | Gall stones, submucosal cyst of the choledochus and gall bladder | Choledochotomy, cholecystectomy | alive | Chait | 1994 |
| 6 | 48 | None | I | None | Resection of the choledochal cyst, cholecystectomy | alive | present case | |

resection of the choledochal cyst. After the drainage operation, the previously dilated intrahepatic bile ducts have been decreasing in size. However, because the choledochal cyst in the present case does not seem to have been caused by pancreaticobiliary maljunction as described above, reflux of the pancreatic juice into the biliary system could not have been a contributing factor for complications such as malignant change, stone formation even in the long-term follow-up. If pancreaticobiliary maljunction had been completely denied, observation would have been a better choice. Conservative therapy with ursodiol or chenodeoxycholic acid may be useful for preventing the stone formation [13]. In addition, endoscopic sphincterotomy may be useful for drainage of the bile *via* the papilla of Vater [14]. Thus, management of the choledochal cyst, when associated with other fibropolycystic disease, requires further discussion.

In conclusion, association of Type I choledochal cyst and polycystic kidney is very rare. In the case of choledochal cyst combined with renal fibropolycystic disease, pancreaticobiliary maljunction may not contribute to the etiology of choledochal cyst. In such cases, management of choledochal cyst is still controversial and requires further discussion.

### *References*

[1] Wechsler, R. L. and Thiel, V. D. (1976). Fibropolycystic disease of the hepatobiliary system and kidneys, *Digestive Diseases*, **21**, 1058–1069.

[2] Summerfield, J. A., Nagafuchi, Y., Sherlock, S., Cadafalch, J. and Scheuer, P. J. (1986). Hepatobiliary fibropolycystic diseases: A clinical and histological review of 51 patients, *Journal of Hepatology*, **2**, 141–156.

[3] Waldron, R., Drumm, J., McCarthy, C. F. and Murphy, B. (1984). Choledochal cysts-report of three cases and review, *Postgraduate Medical Journal*, **60**, 397–399.

[4] Jordan, D., Harpaz, N. and Thung, S. N. (1989). Caroli's disease and adult polycystic kidney disease: a rarely recognized association, *Liver*, **9**, 30–35.

[5] Takehara, Y., Takahashi, M., Naito, M., Kato, T., Nishimura, T., Isoda, H. and Kaneko, M. (1989). Caroli's disease associated with polycystic kidney: its noninvasive diagnosis, *Radiation Medicine*, **7**, 13–15.

[6] Chait, M. M., Rie, J. and Altman, B. (1994). Hepatobiliary fibropolycystic disease in a patient with autosomal dominant polycystic kidney disease, *Gastrointestinal Endoscopy*, **40**, 759–761.

[7] Babbit, D. P. (1968). Congenital choledochal cysts: New etiological concept based on anomalous relationships of the common bile duct and pancreatic bulb, *Annals of Radiology*, **12**, 231–240.

[8] Todani, T., Watanabe, Y., Naruse, M., Tabuchi, K. and Okajima, K. (1977). Congenital bile duct cysts: Classification, operative procedures, and review of thirty-seven cases including cancer arising from choledochal cyst, *American Journal of Surgery*, **134**, 263–269.

[9] Komi, N., Kuramoto, M., Udaka, H., Ikeda, N. and Kuwashima, T. (1981). Congenital biliary dilatation: Cooperative study, *Tokushima Journal of Experimental Medicine*, **28**, 91–95.

[10] Okada, A., Nakamura, T., Higaki, J., Okumura, K., Kamata, S. and Oguchi, Y. (1990). Congenital dilatation of the bile duct in 100 instances and its relationship with anomalous junction, *Surgery Gynecology and Obstetrics*, **171**, 291–298.

[11] O'Neil, J. A. (1992). Choledochal cyst, *Current Problems in Surgery*, **29**, 365–410.

[12] Berenguer, J., Olaso, V., Rayon, M., Gordo, G., Ruiz-Alonso, J., Carrasquer, J. and Baguena, J. (1976). Dilatacion congenita no obstructiva de los conductos biliares intrahepaticos segmentarios (enfermedad de Caroli): presentacion de una observacion y revision de la literatura, *Revista Clinica Espanola*, **140**, 567–577.

[13] Kutz, K., Miederer, S. E. and Baumgartner, G. (1978). Case report: Chenodeoxycholic acid therapy of intrahepatic radiolucent gallstones in a patient with Caroli's syndrome, *Acta Hepato-gastroenterolica*, **25**, 398–401.

[14] Venu, R. P., Geenen, J. E., Hogan, W. J., Dodds, W. J., Wilson, S. W., Stewart, E. T. and Soergel, K. H. (1984). Role of endoscopic retrograde cholangiopancreatography in the diagnosis and treatment of choledochocele, *Gastroenterology*, **87**, 1144–1199.

### COMMENTARY

### Hepatobiliary Fibrocystic Diseases

Hepatobiliary Fibropolycystic diseases do not exist as single entities, but as members of a family [1]. The members are found in various combinations. They consist of polycystic liver, microhamartoma, congenital hepatic fibrosis, congenital intra-hepatic biliary dilatation and choledochal cysts. They are usually inherited. Fibrocystic disease of the kidneys is associated to a variable extent [2]. Embryologically the hepatobiliary abnormalities are thought to stem from ductal plate maldevelopment in different parts of the biliary tree [3]. Malignant change may complicate congenital hepatic fibrosis and choledocysts (which include Caroli's disease) [2].

The classification of the types of choledochal cyst needs to be clarified here in order to fully appreciate the uniqueness of this case report by Hasegawa *et al.*, Alonzo-Lej and coworkers first described three types of choledochal cysts: Type I, cystic dilatation of the common bile duct; Type II, diverticular malformation of the common duct and Type III, choledochocele. The intrahepatic and the extrahepatic biliary tree (apart from the choledocyst) is normal [4]. Todani and associates later added Type IV as multiple cysts, either intrahepatic and extrahapatic or only extrahepatic [5]. Type V are single or multiple intrahepatic cysts [6,7], and when they are associated with hepatic fibrosis, they are referred to as Caroli's disease, as described by Caroli and his coworkers in 1958 [8].

This case report by Hasegawa *et al.*, is on a 48-year old female who had a Type I choledochal cyst which was associated with polycystic kidney disease. Of the five previous reported cases associated with polycystic kidney disease, three had Caroli's disease (Type V) [9–11] and two had Type IV choledochal cysts [12,13].

In contrast to the authors of this case report, we do not believe that it is controversial as how Types I and II choledochal cysts should be treated, no matter whether these cysts are associated with polycystic kidney disease or not. The best treatment for these cysts is cyst excision with Roux-en-y hepaticojejunostomy reconstruction as this provides the best drainage to the biliary system as well as removing the chance of malignant changes in the cyst wall. Roux-en-y choledochocystjejunostomy should be reserved for cases in which technically the cyst is difficult to excise due to inflammation and adhesions caused by repeated cholangitis. The chance that this needs to be done is very small in good hands. Other forms of choledochal cysts, such as choledochocele, intrahepatic cysts and Caroli's disease, are treated as the anatomy dictates and these patients must be followed up on a long term basis.

### *References*

[1] Summerfield, J. A., Nagafuchi, Y., Sherlock, S., Cadafalch, J. and Schener, P. J. (1986). Hepatobiliary fibropolycystic diseases: A clinical and histological review of 51 patients, *Journal of Hepatology*, **2**, 141–156.

[2] Sherlock, S. and Dooley, J. (1997). Cysts and Congenital biliary abnormalities. In: *Diseases of the Liver and Biliary System*, Sherlock, S., Dooley, J, (Eds.), 10th ed. Oxford, Blackwell Science, Ch. 30, pp. 579–591.

[3] D'Agata, I. D. A., Joans, M. M., Peretz-Atayde, A. R., Guay-Woodford, L. M. (1994). Combined cystic disease of the liver and kidney, *Seminars In Liver Disease*, **14**, 215–228.

[4] Alonzo-Lej, F., Revor, W. B. and Pessagno, D. J. (1959). Congenital choledochal cyst, with a report of 2, and an analysis of 94 cases, *Surg. Gynecol. Obstet. Int. Abstr. Surg.*, **108**, 1–30.

[5] Todani, T., Watanabe, Y. and Narusue, M. (1997). Congenital bile duct cysts classification, operative procedures, and review of 37 cases including cancer arising from choledochal cyst, *Am. J. Surg.*, **134**, 263–269.

[6] O'Neil, J. A. (1992). Choledochal cyst, *Curr. Probl. Surg.*, **29**, 365–410.

[7] Taylor, L. A. and Ross, A. J. (1991). Abdominal masses. In: Walker, A. W., Devine, P. R. and Hamilton, J. R. (Eds.). *Pediatric Gastrointestinal Disease*, Philadelphia, B.C. Decker Inc. P. 134.

[8] Caroli, J., Soupalt, R. and Kossakowski, J. (1958). La dilatation polykystique congenitale des voies biliaires intrahepatiques. Essar de classification, *Sem Hop Paris*, **34**, 488–495.

[9] Berenquer, J., Olaso, V., Rayon, M., Gordo, G., Ruiz-Alonso, J., Carrasquer, J. and Baquena, J. (1976). Dilatacion congenita no obstruction de los conductos biliaires intrahepaticos segmentarios (enfermedad de caroli): presentacion de una obsevacion y revision de la literatura. *Revista clinica Espanola*, **140**, 567–577.

[10] Tehara, Y., Takahashi, M., Naito, M., Kato, T., Nishimura, T., Isoda, H. and Kaneko, M. (1989). Caroli's disease associated with polycystic kidney: its noninvasive diagnosis, *Radiation Medicine*, **7**, 13–15.

[11] Jordan, D., Harpaz, N. and Thung, S. N. (1989). Caroli's disease and adult polycystic kidney disease: a rarely recognised association, *Liver*, **9**, 30–35.

[12] Waldron, R., Drumm, J., McCarthy, C. F. and Murphy, B. (1984). Choledochal cyst–report of three cases and review, *Postgraduate Medical Journal*, **60**,

[13] Chait, M. M., Rie, J. and Altman, B. (1994). Hepatobiliary fibropolycystic disease in a patient with autosomal dominant polycystic kidney disease, *Gastrointestinal Endoscopy*, **40**, 759–761. 397–399.

W. Y. Lau and A. K. C. Li
Department of Surgery
The Chinese University of Hong Kong
Prince of Wales Hospital
Shatin, New Territories
Hong Kong

